# Pre-existing systemic inflammation impairs bacterial clearance in the spleen

**DOI:** 10.1186/s40635-026-00865-w

**Published:** 2026-02-04

**Authors:** Katja Hanslin, Paul Skorup, Frida Wilske, Anders Larsson, Eva Tano, Jan Sjölin, Miklos Lipcsey

**Affiliations:** 1 Department of Surgical Sciences/Anesthesiology and Intensive Care Medicine, Uppsala University, Uppsala, Sweden; 2 Section of Infectious Diseases, Department of Medical Sciences, Uppsala University, Uppsala, Sweden; 3 Section of Clinical Chemistry, Department of Medical Sciences, Uppsala University, Uppsala, Sweden; 4 Section of Clinical Bacteriology, Department of Medical Sciences, Uppsala University, Uppsala, Sweden; 5 Hedenstierna Laboratory, Department of Surgical Sciences, Uppsala University, Uppsala, Sweden

**Keywords:** Bacterial clearance, Animal models, Endotoxin, *Escherichia coli*, Mononuclear phagocyte system, Sepsis

## Abstract

**Background:**

Sepsis-induced immunosuppression impairs bacterial clearance and increases mortality. Liver and spleen macrophages are an essential part of the mononuclear phagocyte system and crucial for bacterial elimination. Systemic inflammation hampers bacterial clearance in the liver. We hypothesized that immunosuppression due to systemic inflammation might lead to decreased bacterial clearance by the spleen.

**Methods:**

Anesthetized pigs were subjected to an *E. coli* infusion for three hours in an intensive care setting. The Naive group only received the bacterial infusion (*n* = 10). The ETX group (*n* = 10) received an endotoxin infusion for 24 h, inducing systemic inflammation before the *E. coli* infusion. The Control group (*n* = 3) received saline instead of endotoxin for 24 h to study the effects of anesthesia alone. Bacterial counts and endotoxin levels were analyzed during the *E. coli* infusion. The levels of IL-6 and TNF were analyzed, and the piglets’ physiological response was evaluated.

**Results:**

There was no difference in the bacterial counts in the artery, splenic vein or hepatic vein. However, the splenic venous to arterial bacterial counts ratio at 1–3 h was lower in Naive compared to ETX group (0.54 (0.34–1.07), 0.54 (0.20–0.85), 0.52 (0.21–0.64) vs. 0.77 (0.61–1.06), 0.85 (0.63–0.89), 0.74 (0.53–0.88); *p* < 0.01), but there was no difference in the animals’ blood ex vivo bactericidal capacity. There was no difference in endotoxin clearance in the spleen between the groups. The peak log IL-6 and TNF levels in response to the *E. coli* infusion were higher in the Naive compared to the ETX group (3.40 ± 0.41 vs. 2.94 ± 0.43 pg × mL^−1^ and 3.78 ± 0.68 vs. 2.23 ± 0.36 pg × mL^−1^; *p* < 0.05 and *p* < 0.001, respectively). The respiratory, circulatory and metabolic response to the *E. coli* infusion was dampened in the animals pre-exposed to endotoxin.

**Conclusion:**

The splenic bacterial clearance is impaired by pre-existing systemic inflammation, while differences in endotoxin elimination were not detected. The inflammatory and physiological response to bacteremia is diminished during ongoing inflammation. Since the animals’ ex vivo bactericidal capacity was not affected by pre-existing inflammation, our results suggest that inherent mechanisms in the spleen were involved.

**Supplementary Information:**

The online version contains supplementary material available at 10.1186/s40635-026-00865-w.

## Background

Despite reports of decreasing mortality rates, sepsis is still a major cause of morbidity and death [1, 2]. Early in sepsis, both the pro-inflammatory and anti-inflammatory pathways are activated, but the majority of deaths due to sepsis occur during the following sustained immunosuppressive state [3–5]. Sepsis-induced immunosuppression, characterized by dysfunction in both innate and adaptive immune responses, can lead to inadequate clearance of infectious foci, increased risk of secondary infections, and mortality [5–7]. Sepsis in patients with recent or ongoing inflammatory activation is a highly clinically relevant situation in intensive care units (ICU), bearing similarities to the state of endotoxin tolerance [8, 9]. Tissue macrophages in the liver and spleen are an essential part of the mononuclear phagocyte system (MPS) and are important for bacterial elimination [10]. The spleen is crucial for bacterial clearance from the circulation by its macrophage bactericidal capacity, and studies indicate that the spleen also contributes to bacterial endotoxin elimination [11, 12]. The spleen acts as a filter for opsonized bacteria and can trigger innate and adaptive immune responses to pathogens [13]. Patients with functional or anatomic asplenia have a higher risk of sepsis and overwhelming infections with high mortality rates [13].

In a previous study, we reported that hepatic bacterial clearance is impaired in piglets with pre-existing systemic inflammatory response [14]. It was therefore of interest to investigate the bacterial clearance of the spleen in a similar experimental porcine intensive care model and to assess whether this clearance was affected by an ongoing systemic inflammatory reaction. Thus, our primary endpoint was to study bacterial clearance by the spleen during an intravenous *Escherichia coli* (*E. coli*) infusion in previously healthy piglets and animals with pre-existing systemic inflammatory response. Our secondary endpoint was to investigate splenic endotoxin elimination during an *E. coli* infusion.

## Materials and methods

### Ethical statement

The Animal Ethics Board in Uppsala, Sweden, approved the experiments (Dnr. C155/14 and 08592-2019). The piglets were handled in accordance with the Guide for the Care and Use of Laboratory Animals (EU Directive 2010/63/EU). Minimum Quality Threshold in Preclinical Sepsis Studies (MQTiPSS) guidelines [15] were followed when relevant, and ARRIVE guidelines [16] for reporting were followed.

### Study design

**Fig. 1:**
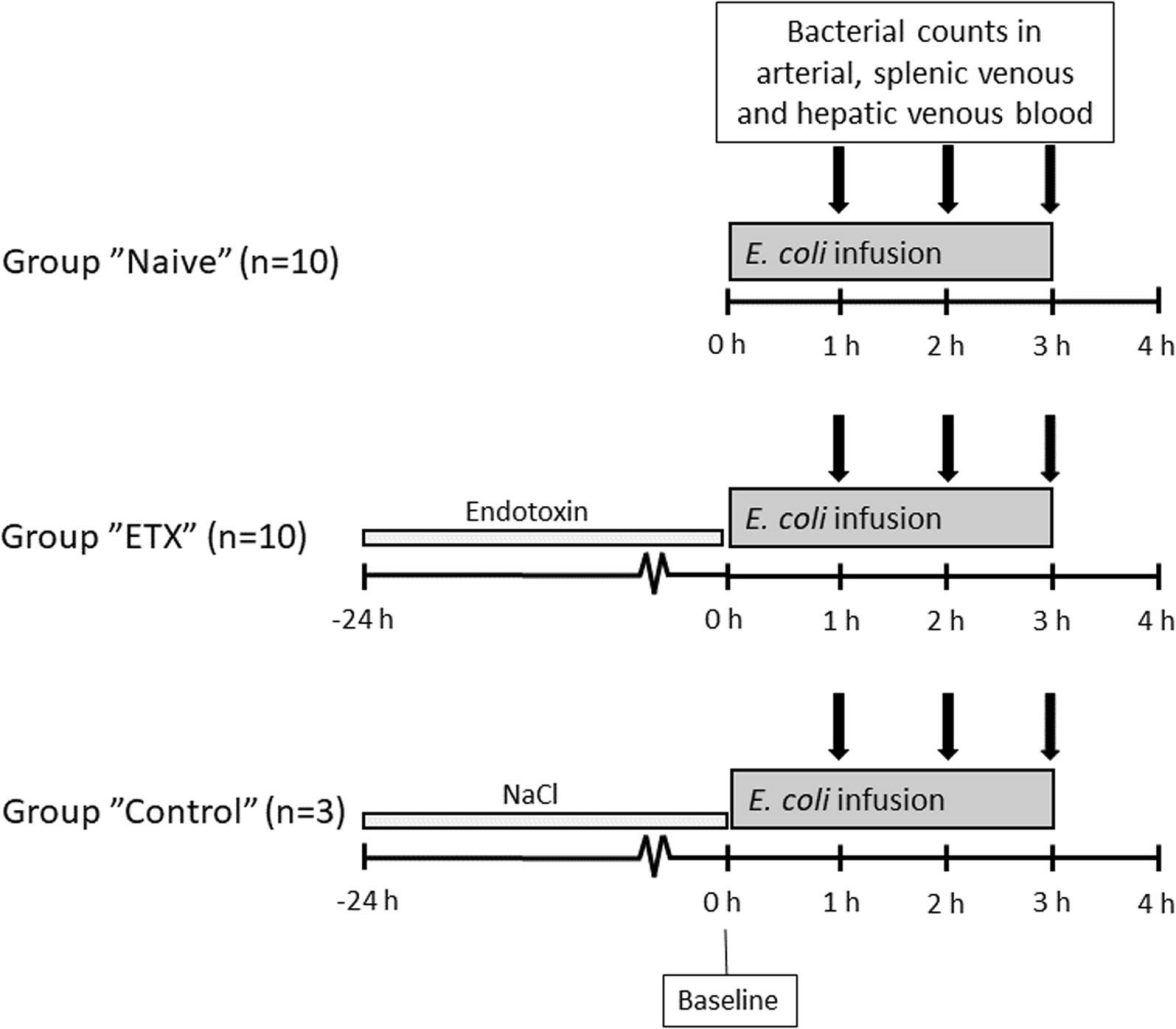
Overview of the study design. All animals were subjected to an intravenous infusion of *E. coli*. Naive group only received the *E. coli* infusion. A continuous infusion of endotoxin was administered in the group “ETX” for 24 h prior to the *E. coli* infusion to induce systemic inflammation. The Control group served as controls for 24 h of anesthesia and intensive care and received saline for 24 h before the *E. coli* infusion. Blood samples for the analysis of bacterial counts in the artery, splenic vein, and hepatic vein were collected hourly during the *E. coli* infusion

The animals were allocated to one of three groups: “Naive” (*n* = 10), “ETX” (*n* = 10) and “Control” (*n* = 3) (Fig. 1). To study the splenic bacterial clearance, all animals were exposed to an intravenous (i.v.) infusion of live *E. coli*. The Naive group only received the *E. coli* infusion. To induce a systemic inflammatory reaction in the ETX group, the animals were subjected to an endotoxin infusion for 24 h before the bacterial challenge. The Control group acted as a control for the effects of 24 h of anesthesia and was given saline instead of endotoxin for 24 h before the bacterial infusion.

### Experimental animals

Norwegian landrace breed piglets and a cross-breed of Swedish landrace breed, Yorkshire, and Hampshire piglets, both 6–8 weeks old and of both sexes, were used for the experiments. The piglets were housed on a farm, transported to the laboratory before the experiments, and had access to food and water ad libitum until one hour before the start of the experiment.

### Preparatory procedures

The animals were anesthetized and mechanically ventilated throughout the experiment, and all preparations were performed under aseptic conditions. The airway was secured with a tracheotomy. A cervical artery was catheterized for pressure monitoring and blood sampling. A central venous line and a Swan–Ganz catheter were both inserted through the right external jugular vein into the superior vena cava and the pulmonary artery, respectively. A catheter was inserted in the splenic vein through a small laparotomy, and another catheter was inserted into a hepatic vein via the left external jugular vein and controlled using fluoroscopy. A urinary catheter was inserted through a minimal vesicostomy. The animals were covered and placed on a heating pad (Operatherm 200W; KanMed, Bromma, Sweden) to decrease heat losses and placed on their right side.

### Experimental procedures

A detailed description of the induction and maintenance of anesthesia, ventilatory settings and fluid administration can be found in Supplementary Information. The animals were continuously monitored for anesthetic depth and signs of pain and distress and treated according to a protocol to maintain vital parameters within preset limits (Table S1, Supplementary Information). All animals received an infusion of live *Escherichia coli* (*E. coli*) through the central venous catheter for three hours. The Naive group received only the *E. coli* infusion. To induce a mild systemic inflammatory response, the ETX group was exposed to an i.v. endotoxin infusion (*Escherichia coli*: 0111:B4; Sigma Chemical, St. Louis, MO) at 0.063 µg × kg × h^−1^ [17] for 24 h, before the *E. coli* infusion. The Control group received saline instead of endotoxin for 24 h prior to the *E. coli* infusion. At the end of the experiment, all animals were culled by i.v. potassium chloride.

### Bacterial preparations and cultures

Details on the preparation of *E. coli* and the method used for bacterial cultures can be found in the Supplementary Information. The *E. coli* (B09-11822 serotype O-rough:K1:H7; Statens Serum Institut, Copenhagen, Denmark), an encapsulated clinical isolate, was used for the experiment. A target bacterial dose of a total of 8.23 log_10_ colony-forming units (CFUs) was infused in the central venous catheter in 3 h. During the *E. coli* infusion, blood samples for bacterial counts were collected every hour from the splenic vein, from the artery and the hepatic vein. Blood samples to determine the ex vivo bactericidal capacity were collected at 0 h, before the start of the bacterial infusion. To determine the pig blood bactericidal capacity, blood was inoculated ex vivo with 10^5^ CFU × mL^−1^ *E. coli* in duplicate at 37 °C. Viable counts were plated hourly for 6 h.

### Measurements

Physiological data were registered at predetermined intervals during endotoxin or saline infusion in the ETX and Control groups, and hourly after the start of the bacterial infusion at 0 h in all groups. Airway pressure values and respiratory volumes were recorded from ventilator readings. Static compliance was calculated using the plateau pressure obtained during a 2-s end-inspiratory hold, and the calculated value, using the corresponding tidal volume, was recorded from the ventilator’s displayed readings. Mean arterial pressure (MAP), mean pulmonary arterial pressure (MPAP), heart rate (HR), and central venous pressure (CVP) were monitored continuously using Philips IntelliVue MX800 or MP50 patient monitor (Philips Healthcare, Eindhoven, The Netherlands). Cardiac output (CO) was measured hourly using the thermodilution technique via the Swan–Ganz pulmonary artery catheter. Urine output was measured, and urine samples were collected at predetermined intervals. Endotoxin levels were analyzed in splenic venous and arterial blood at 0 h (before the start of the bacterial infusion) and at 3 h in the Naive group, and at − 24, 0, and 3 h in the ETX and Control groups. Blood samples for analysis of blood gases, blood cell count, creatinine, tumor necrosis factor (TNF), and interleukin 6 (IL-6) were taken at the start of the experiments, during the endotoxin or saline infusion in the ETX and Control groups, and hourly starting at baseline in all groups. Additional details can be found in the Supplementary Information, including an overview of the blood and urine sampling during the experiments (Table S2).

### Experimental outcomes

As in our previous study on hepatic bacterial clearance [14], splenic bacterial clearance was assessed by the splenic venous to arterial bacterial counts ratio, and splenic endotoxin elimination by the splenic venous to arterial endotoxin levels ratio during the *E. coli* infusion. The inflammatory response to the *E. coli* infusion was measured by intergroup comparison of TNF and IL-6 levels, and the physiological response by measuring physiological parameters, blood samples, and blood gases analyses.

### Statistical methods and calculations

With a standard deviation of 0.2 for the primary endpoint in the Naive group, a power of 0.8, a two-sided alpha of 0.05, and a detectable difference of 30%, a minimum of 7 animals in each group were required. The statistical analysis was performed according to a predetermined plan. The bacterial counts were adjusted for variations in piglet weight and infused bacterial dose. Data were tested for normality. Data with log-normal distribution were log-transformed. All values are expressed as mean ± SD or median (IQR) as appropriate, unless otherwise stated. For normally distributed data, Student’s *t-test* or one-way ANOVA was used for intergroup comparisons. ANOVA for repeated measurements with type III sums of squares for significance testing was used to assess group differences. For non-normally distributed data, the Mann–Whitney *U* test was performed for intergroup comparisons and Spearman’s rank test for correlations. To assess the group difference in animals’ ex vivo bactericidal capacity, we performed a time to level below limit-of-detection analysis for viable bacteria with a Log-rank test. All analyses were performed using Statistica™ software (version 14, StatSoft Inc., Tulsa, OK, USA), and a *p*-value of < 0.05 was considered significant. The Control group was added to study the effects of 24 h anesthesia, and the results are presented in graphs, but the group was not included in the primary analyses.

## Results

The initial characteristics of the animals are depicted in Table 1. All animals survived throughout the experiment. The pro-inflammatory cytokines IL-6 and TNF peaked two hours after the start of the endotoxin infusion in the ETX group as a sign of systemic inflammatory response (Fig. 5).

**Table 1:**
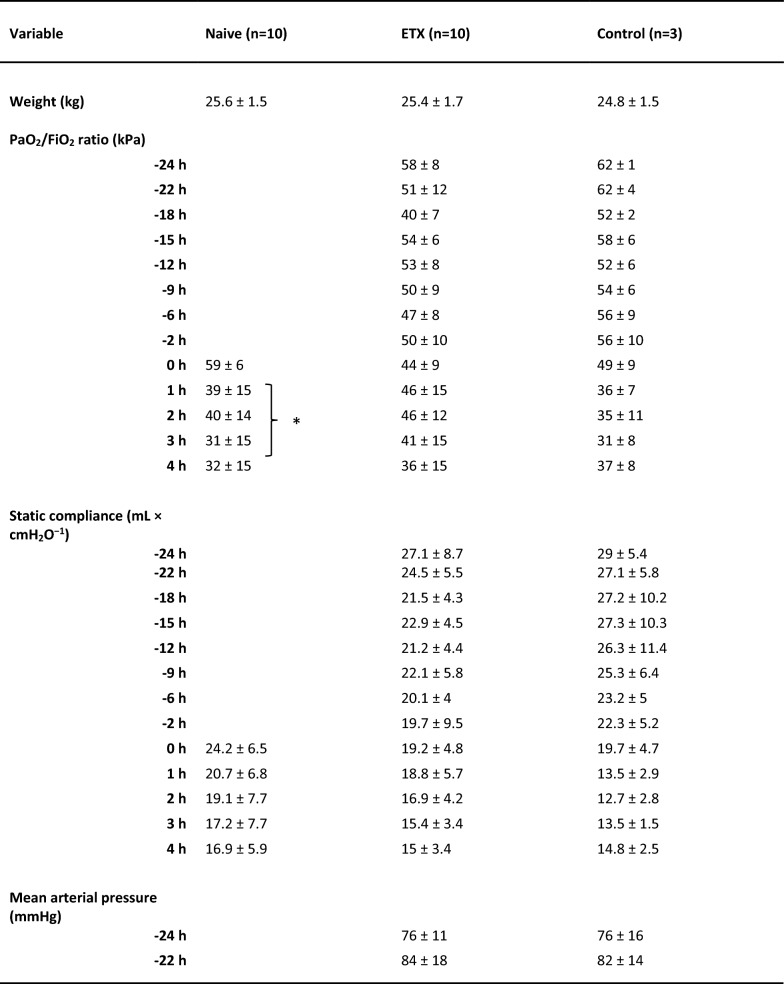

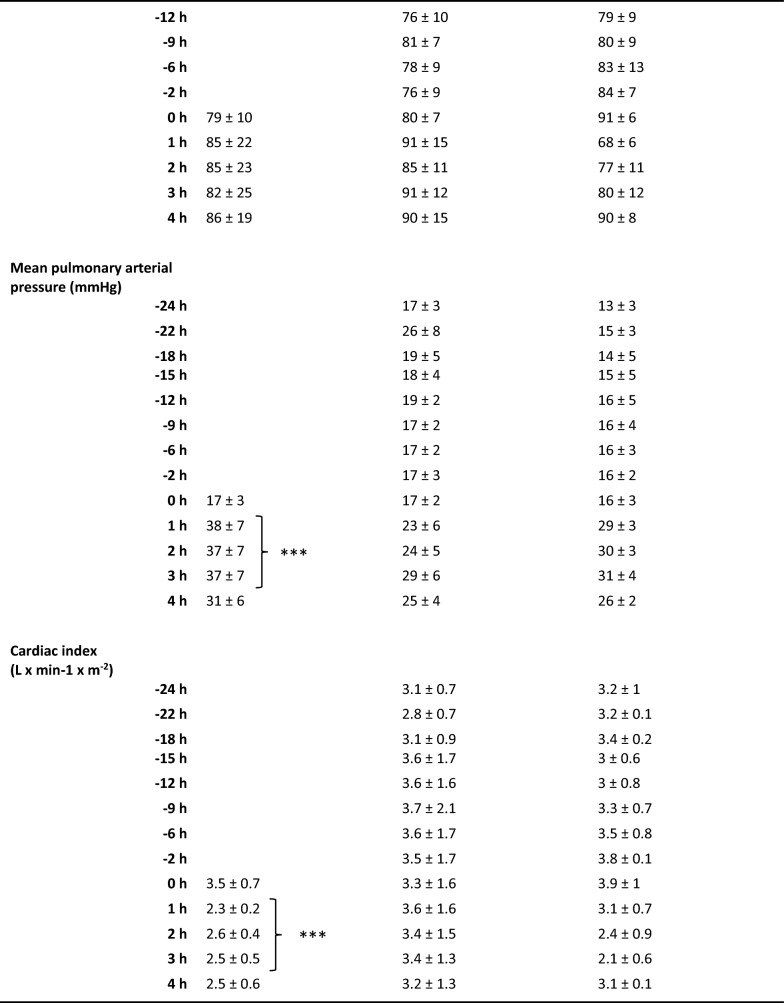

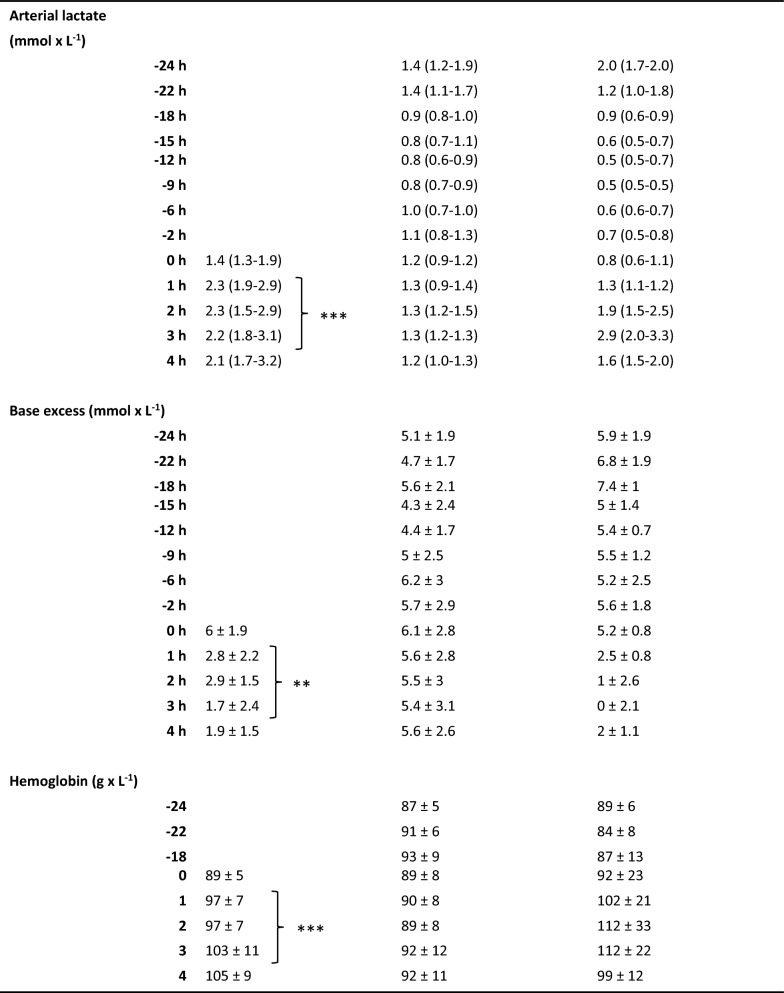

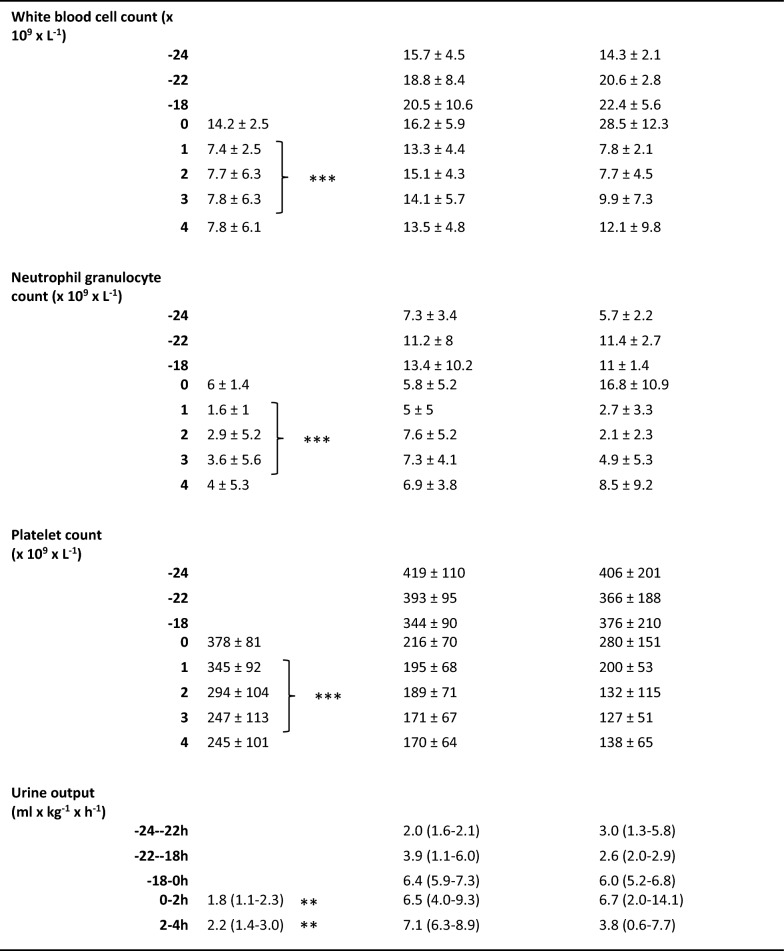

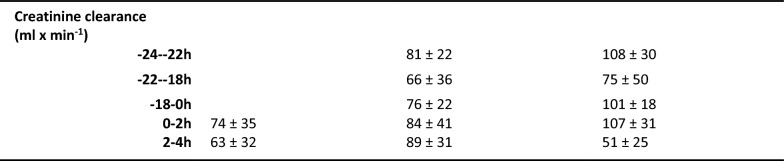
Animals’ weight and physiological variables at baseline and during the experiment. Values are expressed as mean ± SD, or as median (IQR)

An *E. coli* infusion was administered to all animals for 3 h starting at 0 h. The Naive group only received the *E. coli* infusion. The ETX group was given a continuous infusion of endotoxin for 24 h prior to the bacterial infusion. The Control group received saline instead of endotoxin for 24 h. Data were log-transformed when appropriate for the statistical analysis, but all data are presented in non-log form. The difference between the Naive and ETX group 1–3 h was assessed with ANOVA for repeated measurements with type III sums of squares for significance testing, except for urine output where Mann–Whitney was used for intergroup comparison. *p < 0.05, **p < 0.01, ***p < 0.001 for differences between Naive and ETX groups

### Splenic bacterial clearance

**Fig. 2:**
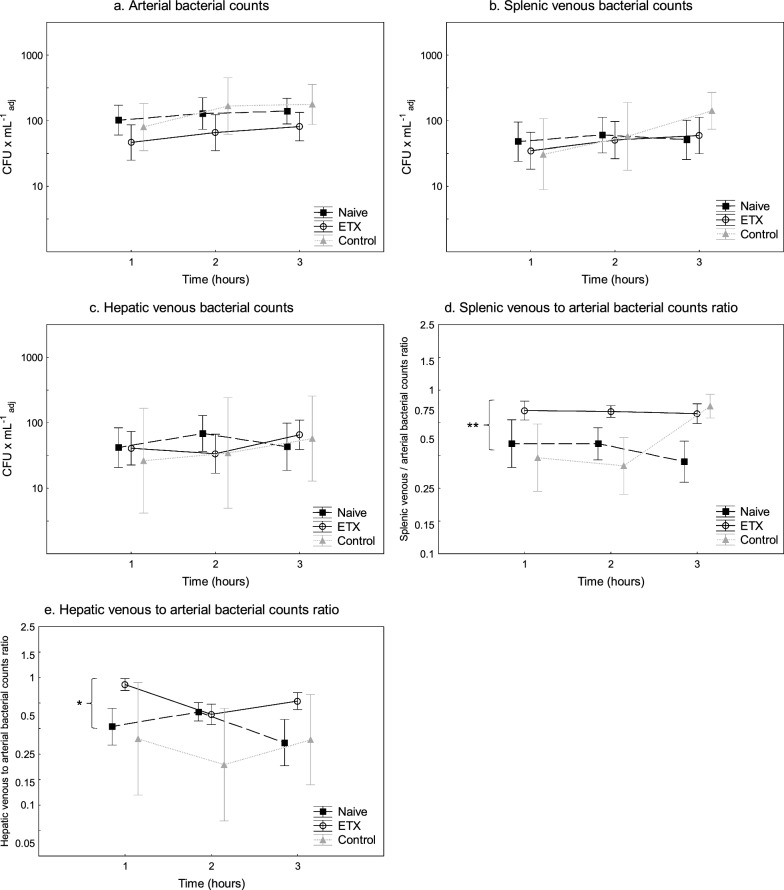
Bacterial counts in the artery, splenic vein, and hepatic vein during the *E. coli* infusion (**a**–**c**). The ratio of splenic venous to arterial bacterial counts (**d**) and the ratio of hepatic venous to arterial bacterial counts (**e**) during the *E. coli* infusion. An *E. coli* infusion was administered to all animals for 3 h starting at 0 h. The Naive group only received the *E. coli* infusion. The ETX group was given a continuous infusion of endotoxin for 24 h prior to the bacterial infusion. The Control group received saline instead of endotoxin for 24 h. **p < 0.01 for the difference between Naive and ETX groups at 1–3 h. Values are expressed as mean ± SEM (standard error of the mean)

The administered *E. coli* doses were similar in the Naive and ETX groups (8.68 ± 0.11 vs. 8.74 ± 0.11 log_10_ CFU). There were no differences in the arterial, splenic venous, or hepatic venous bacterial counts, whereas the splenic venous to arterial bacterial counts ratio was lower, indicating a higher clearance in the Naive group compared to the ETX group (*p* < 0.01; Fig. 2). Individual bacterial counts taken simultaneously in the artery and splenic vein are shown in Figure S1 in the Supplementary Information.

### Blood ex vivo bactericidal capacity

**Fig. 3:**
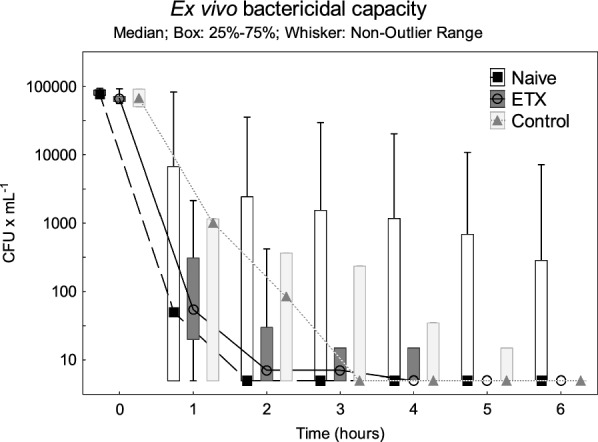
Bacterial counts exhibiting blood ex vivo bactericidal capacity in Naive (*n* = 10), ETX (*n* = 10), and Control (*n* = 3) groups. An *E. coli* infusion was administered to all animals for 3 h starting at 0 h. The Naive group only received the *E. coli* infusion. The ETX group was given a continuous infusion of endotoxin for 24 h prior to the bacterial infusion. The Control group received saline instead of endotoxin for 24 h. Values are expressed as median (IQR)

There was no difference between the Naive and ETX groups in the animals’ ex vivo bactericidal capacity (*p* = 0.67 log-rank test; Fig. 3). There was no correlation between the ex vivo bactericidal capacity in blood and the splenic bacterial elimination (*ρ* = 0.07, − 0.05, and 0.24; Spearman correlation between ex vivo at 1 h and the ratio of splenic venous to arterial bacterial counts ratio at 1, 2, and 3 h, respectively).

### Hepatic bacterial clearance

A post hoc analysis of bacterial clearance in the liver was conducted. Bacterial concentrations in the hepatic vein are shown in Fig. 2. The hepatic venous to arterial bacterial counts ratios were lower in the Naive group compared to the ETX group during the *E. coli* infusion (*p* < 0.05; Fig. 2).

### Splenic endotoxin clearance

**Fig. 4:**
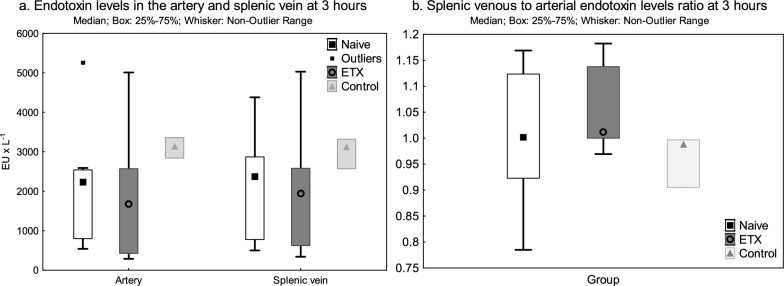
Endotoxin levels in the artery and splenic vein at 3 h during the *E. coli* infusion (**a**) and splenic venous to arterial endotoxin levels ratio during the *E. coli* infusion at 3 h (**b**). An *E. coli* infusion was administered to all animals for 3 h starting at 0 h. The Naive group only received the *E. coli* infusion. The ETX group was given a continuous infusion of endotoxin for 24 h prior to the bacterial challenge. The Control group received saline instead of endotoxin for 24 h. Values are expressed as median (IQR)

The endotoxin levels were below detection limit in the artery and the splenic vein at − 24 h in the ETX group. The endotoxin levels in the artery and splenic vein at 0 h were primarily below detection limit in the Naive group (< 50 (< 50−  < 50) EU × L^−1^ for both), with only a few animals exhibiting increased endotoxin levels, and slightly elevated in the ETX group during ongoing endotoxin infusion (121 (59–180) and 189 (109–201) EU × L^−1^, respectively). There were no differences in endotoxin levels in the artery or the splenic vein, and no difference in the splenic vein to arterial endotoxin levels ratio between the groups at 3 h (Fig. 4).

### Cytokine response

**Fig. 5:**
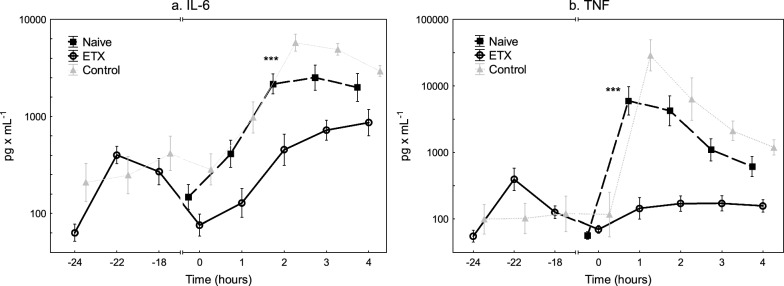
The levels of interleukin 6 (IL-6) (**a**) and tumor necrosis factor (TNF) (**b**) during the experiment. An *E. coli* infusion was administered to all animals for 3 h starting at 0 h. The Naive group only received the *E. coli* infusion. The ETX group was given a continuous infusion of endotoxin for 24 h prior to the bacterial infusion. The Control group received saline instead of endotoxin for 24 h. Values are expressed as mean ± SEM (standard error of the mean). ***p < 0.001 for the difference between the Naive and ETX group at 1–3 h

The IL-6 and TNF levels were higher in Naive compared to the ETX group during the *E. coli* infusion (Fig. 5, *p* < 0.001 for both). The IL-6 and TNF levels peaked earlier, and the peak levels were higher in the Naive compared to the ETX group (*p* < 0.05 and *p* < 0.001, respectively).

### Physiological response

The PaO_2_/FiO_2_ ratio was lower in the Naive vs. ETX group (*p* < 0.05; Table 1), but no difference in static compliance between the groups could be detected at 1–3 h, during the *E. coli* infusion. MPAP was higher, and cardiac index (CI) was lower, in the Naive vs. ETX group (*p* < 0.001 for both), but there was no difference in MAP, or total dose of norepinephrine used during the bacterial infusion (23.9 (0–132.8) vs. 16.5 (13.0–30.9) µg × kg^−1^). The Naive group received fluid boluses during the bacterial infusion to maintain CI, while the ETX group did not (0 (0–390) vs. 0 (0–0) mL; *p* < 0.05). Arterial lactate levels were higher and base excess (BE) was lower in the Naive group compared to the ETX group (*p* < 0.001 and *p* < 0.01, respectively). Hemoglobin and platelet levels were higher but white blood cell and neutrophil granulocyte counts were lower in the Naive vs. ETX group (*p* < 0.001 for all). Urine output was lower in Naive compared to ETX group (*p* < 0.01), whereas no difference in creatinine clearance between the groups could be demonstrated.

## Discussion

The present study demonstrates that splenic bacterial clearance is impaired by pre-existing systemic inflammatory response, while differences in endotoxin elimination could not be detected. The bacterial elimination effect was not seen in ex vivo experiments, suggesting that this is an inherent effect of the spleen and not mediated by circulating factors in the blood. The pro-inflammatory cytokine response and the physiological response to an *E. coli* infusion are attenuated by pre-existing systemic inflammation.

Our findings indicate that systemic inflammation impairs splenic bacterial clearance. The current findings are consistent with our previous results regarding the liver, where a similar conclusion was reached [14]. Normally, the liver and the spleen together stand for the rapid elimination of bacteria from the circulation [18], but the mechanisms behind their bacterial clearances are different. The spleen lacks afferent lymphatic vessels and therefore all antigens, such as whole bacteria, pathogen-associated molecular patterns (PAMPs), and cells are transported to the spleen via the systemic circulation [19]. Phagocytosis and other innate immune responses are elicited as macrophage pattern recognition receptors (PRRs), such as the toll-like receptors (TLRs), are activated by PAMPs [19]. Blood pools in the red pulp through the splenic artery, and macrophages in the red pulp and perifollicular zone filter the blood from circulating bacteria before passing through the venous sinuses back to the circulation [19, 20]. Conversely, in the liver, bacteria are removed directly from the circulation by Kupffer cells within the hepatic sinusoids without any filtration or trapping [21]. Although a higher uptake of bacteria in the spleen has been reported [22], the liver, given its size, has a high capacity, indicating that both organs contribute significantly to bacterial clearance from the blood.

Bacterial elimination during endotoxin tolerance has been investigated in rodents with conflicting results [23–27], most of them, however, demonstrating an increased bacterial killing. Similarly, using the same porcine intensive care sepsis model as in the present study, enhanced bacterial killing has been observed in animals pre-exposed to a 24-h endotoxin infusion [22]. Bacterial clearance from the blood has also been investigated in one study in rabbits and, in similarity with our results, a decreased clearance was observed [27]. The mechanisms behind a lower clearance in the presence of increased bacterial killing in piglets pre-exposed to endotoxin are not clear. However, considering the filtration component of the splenic process, one explanation for the increased venous to arterial ratio might be a non-killing mechanism, such as an augmented leakage of bacteria from the splenic red pulp to the venous system. This may be caused by an inflammation-induced increased permeability affecting the venous interendothelial slits that under normal conditions are supposed to impede rigid bacteria while allowing deformable blood cells to reenter the circulation. The clinical significance of this is unclear, but seems limited in the absence of an increased arterial concentration in the ETX group.

The blood ex vivo bactericidal capacity accounts for the combined bactericidal activity of, e.g., neutrophil granulocytes, the complement system, and host defense antimicrobial proteins present in peripheral blood [28–30]. Preconditioning with endotoxin has been shown to increase bacterial killing of neutrophils in part through the increased formation of neutrophil extracellular traps (NETs) [31]. In the current investigation, the piglets’ ex vivo bactericidal capacity was similar in both groups, albeit with large variations and without any correlation to the splenic bacterial clearance, indicating that the differences in splenic clearances were not caused by changes in the blood bactericidal capacity.

In our previous study on hepatic bacterial clearance, bacteria were infused into the portal vein leading to high portal venous bacterial counts of around 1500 CFU_adj_ × mL^−1^ [14]. In the current study, bacteria were administered via a central venous catheter, and assuming that the spleen is the only organ in the splanchnic region to eliminate bacteria, the following concentration in the portal vein will be modestly lower than that in the artery, thus being around 100 CFU_adj_ × mL^−1^. At the high portal concentration, the reduction in bacterial count was ≈ 1 log_10_, whereas in the present study with a similar setup, this was ≈ 0.3 log_10_, corresponding to reductions of approximately 90% and 50%, respectively. This suggests that hepatic bacterial clearance is not saturable at the bacterial concentrations in this experimental setup, and might even increase at higher preload. Among the individual splenic clearance values, shown in Supplemental Fig. 1, there are no signs neither of a saturable process nor an increased killing at higher concentration within the preload range of the current study. The liver has an important role as a gatekeeper, preventing bacteria that have managed to cross the intestinal barrier from entering the systemic circulation. The liver’s ability to augment bacterial clearance with increasing preload, e.g., in the context of acute intestinal injury, may be an essential attribute.

The liver and spleen are also essential for endotoxin removal from the circulation [32, 33]. The impact of systemic inflammation on splenic endotoxin clearance is unexplored. In contrast to bacterial elimination, the spleen did not follow the liver with a reduction of the endotoxin elimination in animals with an endotoxin-induced pre-existing inflammatory response [14]. With the current setup, we could not detect endotoxin removal across the spleen in either group. As the major organ for endotoxin clearance is the liver [34], the role of the spleen is either not significant enough to be detected by our study or the elimination capacity was saturated by high endotoxin concentrations.

The inflammatory and physiological response to the bacterial challenge was attenuated by pre-exposure to endotoxin, seen as lower TNF and IL-6 levels, and with ongoing organ support only mild alterations in circulatory and metabolic parameters. Similar findings have been found in previous studies by us and others [14, 30, 35, 36], consistent with the development of endotoxin tolerance. Thus, impaired clearance of bacteria by the spleen during systemic inflammation could be a sign of organ dysfunction, the clinical significance of which is unclear.

### Strengths and limitations

To our knowledge, this is the first study to directly compare splenic bacterial clearance in healthy animals and animals with an ongoing inflammatory response. Our large animal model has several advantages compared to rodent and in vitro models. The size of the juvenile pig allows for repeated blood sampling and monitoring. Interventions currently used in the ICUs may all affect the inflammatory response [37–39] and are incorporated into our intensive care animal model. Also, the porcine immune system closely resembles that of humans [40] and the circulation of the pig has been suggested to be most similar to that of humans among non-primates [41]. The two-hit model used has similarities to the clinically relevant situation in intensive care units with sepsis following a non-infectious systemic inflammatory response, such as pancreatitis, trauma or major surgery. The use of the splenic venous to arterial bacterial and endotoxin counts ratio as the primary endpoint analysis has the advantage of taking varying arterial concentrations into consideration, and at the same time being most susceptible to differences if bacterial and endotoxin elimination follow first-order kinetics.

Yet, our study has limitations. Our model uses a low dose of continuous endotoxin infusion to elicit a systemic inflammatory response with a predictable course [17, 42]. A continuous intravenous infusion of a predetermined dose of live *E. coli* is used to induce sepsis in our model, a rarely relevant clinical setting, but has the advantage of eliciting a predictable and reproducible response in the animals suitable for the objectives in our study. The *E. coli* infusion in the current study results in hypodynamic circulation in previously healthy animals, and in hyperdynamic circulation in animals pre-exposed to endotoxin. The regional blood flow changes in sepsis vary, even within the splanchnic circulation [43]. Splenic blood flow measurements were not performed. This could have been of interest if the bacterial uptake processes had been saturable. However, as discussed above, this appears not to be the case. Furthermore, instrumentation of the splenic artery to attach flow probes seemed in pilot experiments to lead to impairment of the flow that could have affected the results.

## Conclusions

A pre-existing systemic inflammatory response leads to impaired bacterial clearance in the spleen, and a diminished cytokine and physiological response to the bacterial infusion. Splenic endotoxin clearance was not shown to be affected by the ongoing inflammatory response in this study.

## Supplementary Information

Additional file 1.

## Data Availability

Most data generated or analyzed during this study are included in this published article (and its supplementary information files). The remaining datasets used and/or analyzed during the current study are available from the corresponding author upon reasonable request.

## Abbreviations

BE: Base excess
CFU: Colony-forming unit
CI: Cardiac index
CO: Cardiac output
CVP: Central venous pressure
*E. coli*: *Escherichia coli*
EU: Endotoxin unit
FiO_2_: Fraction of inspired oxygen
HR: Heart rate
i.v.: Intravenous
ICU: Intensive care unit
IL-6: Interleukin-6
MAP: Mean arterial pressure
MPAP: Mean pulmonary arterial pressure
MPS: Mononuclear phagocyte system
MQTiPSS: Minimum Quality Threshold in Preclinical Sepsis Studies
NET: Neutrophil extracellular traps
PAMPs: Pathogen-associated molecular patterns
PRRs: Pattern recognition receptors
TLR: Toll-like receptor
TNF: Tumor necrosis factor
