# Supplementary material for: Pre-existing systemic inflammation impairs bacterial clearance in the spleen
Source: Intensive Care Med Exp. 2026 Feb 4;14:11. doi: 10.1186/s40635-026-00865-w (PMC12873003; doi:10.1186/s40635-026-00865-w)
Supplement: Supplementary file 1 — Additional file 1. [file 40635_2026_865_MOESM1_ESM.docx]

# Supplementary Information

[Supplementary Information 1](#_Toc220832950)

[Anesthesia, ventilatory settings and fluid administration 2](#_Toc220832951)

[Bacterial preparations and cultures 2](#_Toc220832952)

[Experimental protocol 3](#_Toc220832953)

[Measurements 4](#_Toc220832954)

[Overview of blood and urine sampling during the experiment 5](#_Toc220832955)

[Abbreviations 5](#_Toc220832956)

[References 6](#_Toc220832957)

[Figure legends 6](#_Toc220832958)

## Anesthesia, ventilatory settings and fluid administration

General anesthesia was induced by injecting a mixture of tilétamin-zolazepam 6 mg x kg^-1^ and xylazine 2.2 mg x kg^-1^ intramuscularly. A bolus dose of morphine 20 mg and ketamine 100 mg was given before the surgical preparation and the animals were mechanically ventilated using a Servo I or Servo U (Maquet Critical Care, Stockholm, Sweden). Initial respiratory settings were: respiratory rate 25 x min^-1^, inspiratory-expiratory ratio 1:2, inspired oxygen fraction (FiO_2_) 0.3, positive end-expiratory pressure (PEEP) 5 cmH_2_O and tidal volume (VT) 9 mL x kg^-1^. VT was adjusted before the start of the protocol to result in an arterial pressure of carbon dioxide (PaCO_2_) of 5.0-5.5 kPa. Anesthesia was maintained with continuous infusions of sodium pentobarbital 8 mg x kg x h^-1^ and morphine 0.26 mg x kg x h^-1^ dissolved in 2.5 % glucose, and rocuronium bromide 2.5 mg x kg x h^-1^. The dose of rocuronium bromide was adjusted if shivering was detected. Acetated Ringer’s solution was administered; initially as a bolus of 20 mL x kg^-1^ after induction of anesthesia, and thereafter 2 mL x kg x h^-1^. During surgical preparations, the ETX and Control groups received 750 mg cefuroxime intravenously (i.v.).

## Bacterial preparations and cultures

The *E. coli* was harvested from a frozen isolate, reinoculated on a cysteine lactose electrolyte deficient (CLED) plate and cultured overnight in an incubation chamber at 37 °C. The *E. coli* were grown to a logarithmic growth phase in a lysogeny broth medium according to Miller (LB) (VWR, Spånga, Sweden) two hours before the start of the experiment. The LB broth and bacteria suspension were then centrifuged at 6000 rpm at 22°C for two minutes to enable LB removal, and the remaining bacteria were resuspended in phosphate-buffered saline (PBS). The concentration of the bacterial suspension was determined using spectrophotometry and adjusted when needed. The bacterial infusate was replaced hourly to ensure that the bacteria remained in a logarithmic growth phase. To determine the number of bacteria in the infusate, the *E. coli* solution was cultured on CLED plates after completing the bacterial infusion. To determine bacterial counts in blood, 0.1 mL of blood from the splenic vein, hepatic vein and artery was plated in triplicate on CLED plates every hour during the bacterial infusion. The *E. coli* was identified by colony morphology and the detection limit for growth of bacteria was 5 CFU x mL^-1^. To account for variations in bacterial exposure and piglets’ size, measured bacterial concentrations were adjusted by dividing by the bacterial dose administered and the animal's body weight.

## Experimental protocol

| **Parameter** | **Threshold value for intervention** | **Interventions** |
| --- | --- | --- |
|  |  |  |
| PaO_2_ | <10 kPa first time | Increase FiO_2_ to 0.6 |
| PaO_2_ | <10 kPa thereafter | 1. Increase FiO_2_ to the next level: 0.6🡪0.8 🡪1.0, and |
|  |  | 1. Increase PEEP to the next level: 5🡪8🡪10🡪14 cmH_2_O, and |
|  |  | 1. Lung recruitment maneuver*^a^* |
| PaO_2_ | >30 kPa | Decrease FiO_2_ to the previous level: 1.0🡪0.8🡪0.6🡪0.3 |
| PaCO_2_ | >6.5 kPa | Increase V_T_ with 10 %, to maximum 15 mL x kg^-1^ |
| PaCO_2_ | <4.5 kPa | If the respiratory rate is ≤25 breaths per minute, decrease V_T_ with 10 %. If the respiratory rate is >25, decrease it with 10 %. |
| MAP | MAP = MPAP (<90 min after baseline or start of *E. coli*-infusion) | Single dose of 40 µg norepinephrine i.v. |
| MAP and/or CO | MAP <60 mmHg (>90 min after baseline or start of *E. coli*-infusion) and/or CO<2.0 L x min^-1^. | Start norepinephrine infusion (20 µg x mL^-1^) 5 mL x h^-1^. If ongoing norepinephrine infusion, increase rate one step: 5🡪10🡪20🡪40 mL/h |
| MAP | MAP = MPAP (>90 min after baseline or start of *E. coli*-infusion) | 1. Single dose of 20 µg norepinephrine i.v., and 2. Start norepinephrine infusion (20 µg x mL^-1^) 5 mL x h^-1^. If ongoing norepinephrine infusion, increase rate one step: 5🡪10🡪20🡪40 mL/h, and 3. Fluid bolus with acetated Ringer’s solution 15 mL x kg^-1^ |
| MAP | MAP >100 mmHg | If ongoing norepinephrine infusion (20 µg x mL^-1^), decrease rate one step: 40🡪20🡪10🡪5🡪0 mL x h^-1^ |
| Blood Glucose | <4.0 mmol/L | Give 20 mL of 30 % glucose solution i.v. |

Table S1. PaO_2_ = arterial partial pressure of oxygen, FiO_2_ = inspired fraction of oxygen, PEEP = positive end expiratory pressure, V_T_ = tidal volume, MAP = mean arterial pressure, CO = cardiac output, MPAP = mean pulmonary arterial pressure. *^a^* PEEP was increased stepwise until a peak pressure of 35 cmH_2_O was reached. At this point, an inspiratory hold was performed for 10 s. The PEEP was thereafter stepwise decreased to the PEEP defined by the protocol. In case MAP decreased to the level of the MPAP, the lung recruitment maneuver was aborted.

## Measurements

Circulatory and respiratory variables, as well as creatinine clearance, were calculated using standard formulae [1]. For each cardiac output measurement, a bolus of cold saline was injected into the proximal port of the Swan-Ganz catheter, and the resulting change in blood temperature was detected by a thermistor located at the catheter tip. CO values were automatically calculated by the monitor and recorded from the display (Philips IntelliVue MX800 or MP50 (Philips Healthcare, Eindhoven, The Netherlands)). A mean of three measurements was used. Plasma endotoxin was analyzed in heparinized plasma with the chromogenic Limulus amebocyte lysate assay (LAL) (Endochrome-K; Charles River Endosafe, Charleston, SC, USA), and the limit of detection (LOD) in plasma was 0.5 EU x mL^-1^. Blood from a cervical artery and a pulmonary artery were analyzed for pH, gas tensions (PaO_2_, PaCO_2_), oxygen saturation, lactate, base excess and hemoglobin on an ABL^TM^ 800 and a Hemoximeter ^TM^ OSM-3 (Radiometer, Brønhøj, Denmark). Full blood count was analyzed on a Sysmex XN (Sysmex, Kobe, Japan). Porcine-specific sandwich enzyme-linked immunosorbent assays (ELISA; DY690B [TNF] and DY686 [IL-6], R&D Systems, Minneapolis, MN, USA) were used to measure plasma TNF and IL-6, and the LOD in EDTA plasma was 30 pg x mL^-1^ for TNF and 40 pg x mL^-1^ for IL-6. The ELISAs had total coefficients of variation of approximately 6 %. Creatinine in urine and plasma was measured with enzymatic creatinine reagents (8 L24, Abbott Laboratories, Abbott Park, IL, USA) on a BS380 instrument (Mindray, Shenzhen, China).

## Overview of blood and urine sampling during the experiment

| **Analysis** | **Sample type** | **Location** | **Naive group** | **ETX and Control groups** |
| --- | --- | --- | --- | --- |
| Bacterial cultures | Blood | Artery | 1, 2, 3 h | 1, 2, 3 h |
|  |  | Splenic vein | 1, 2, 3 h | 1, 2, 3 h |
|  |  | Hepatic vein | 1, 2, 3 h | 1, 2, 3 h |
| *Ex vivo* bactericidal capacity | Blood | Artery | 0 h  Thereafter viable count from this sample every hour for 6 h | 0 h  Thereafter viable count from this sample every hour for 6 h |
| Endotoxin | Blood | Artery | 0, 3 h | -24, 0, 3 h |
|  |  | Splenic vein | 0, 3 h | -24, 0, 3 h |
| IL-6, TNF | Blood | Artery | 0-4 h hourly | -24, -22, -18, 0-4 h hourly |
| Blood cell count, creatinine | Blood | Artery | 0-4 h hourly | -24, -22, -18, 0-4 h hourly |
| Creatinine | Urine | Urine collection during intervals | 0-2 h, 2-4 h | -24 - -22h, -22 - -18 h, -18 - 0 h, 0-2 h, 2-4 h |

Table S2. Overview of blood and urine samples taken during the experiment. An *E. coli* infusion was administered to all animals for 3 h starting at 0 h. The Naive group only received the *E. coli* infusion. The ETX group was given a continuous infusion of endotoxin for 24 h prior to the bacterial infusion. The Control group received saline instead of endotoxin for 24 h. h = hours, IL-6 = interleukin 6, TNF = tumor necrosis factor.

## Abbreviations

CLED Cystine Lactose Electrolyte Deficient

ELISA Enzyme-Linked Immunosorbent Assay

FiO_2_ Inspired oxygen fraction

IL-6 Interleukin 6

LAL Limulus amebocyte lysate assay

LB Lysogeny Broth

LOD Limit of Detection

PaCO_2_ Arterial pressure of carbon dioxide

PBS Phosphate-Buffered Saline

PEEP Positive End-Expiratory Pressure

PBS Phosphate-Buffered Saline

PEEP Positive End-Expiratory Pressure

rpm revolutions per minute

TNF Tumor necrosis factor

VT Tidal Volume

## References

1. Irwin, R.S. and J.M. Rippe, *Irwin and Rippe's intensive care medicine*. 7th ed. ed. 2012, Philadelphia, Pa. ; London: Wolters Kluwer/Lippincott Williams & Wilkins.

## Figure legends

**Figure S1.** *E. coli* concentrations in arterial and splenic venous blood during the bacterial infusion in the individual cultures in the Naive (a) and the ETX (b) group. Each case represents cultures from the artery and the splenic vein taken simultaneously during the *E. coli* infusion. Blood samples in the splenic vein could not be collected in one animal during 2-3 hours because of technical difficulties in obtaining the sample.
